# KIF2C is a Biomarker Correlated With Prognosis and Immunosuppressive Microenvironment in Human Tumors

**DOI:** 10.3389/fgene.2022.891408

**Published:** 2022-05-24

**Authors:** Xiuyuan Zhang, Yiming Li, Pengbo Hu, Liang Xu, Hong Qiu

**Affiliations:** Department of Oncology, Tongji Hospital, Tongji Medical College, Huazhong University of Science and Technology, Wuhan, China

**Keywords:** KIF2C, pan-cancer, tumor microenvironment, immunotherapy, biomarker

## Abstract

Kinesin superfamily member 2C (KIF2C) is an essential regulator of the cell cycle and its aberrant expression can promote tumor progression. However, the mechanism of KIF2C in pan-cancer is unclear.Data were obtained from public databases, including The Cancer Genome Atlas (TCGA), UALCAN, TIMER and CellMiner. The data came from public databases such as The Cancer Genome Atlas (TCGA), UALCAN, TIMER, and CellMiner. We analyzed the correlation of KIF2C with expression, prognosis, tumor mutation burden (TMB), microsatellite instability (MSI), mismatch repairs (MMR), immune infiltration and anticancer drug sensitivity by R language.KIF2C was highly expressed in several tumors and correlated with poor prognosis. KIF2C expression was significantly correlated with TMB, MSI, MMRs, and immune checkpoint genes, and with the level of immune cell infiltration such as tumor-associated macrophage (TAM), cancer-associated fibroblasts (CAFs), myeloid-derived suppressor cells (MDSCs) and Tregs. The GO and KEGG results suggest that KIF2C is involved in immune regulation in addition to cell cycle regulation.In addition, KIF2C is associated with DNA methylation, m6A modifications and m7G modifications. Our data suggest that KIF2C is a prognostic biomarker linked to immunosuppression, targeting KIF2C may improve the outcome of immunotherapy. Our findings indicate that KIF2C is a prognostic biomarker associated with immunosuppression, and that targeting KIF2C may improve the outcome of immunotherapy.

## Introduction

The Kinesin-13 Family, which includes KIF2A, KIF2B, KIF2C, and KIF24, is engaged in mitotic regulation (Lucanus and Yip, 2018). KIF2C (Kinesin superfamily member 2C) is the most characteristic member of the Kinesin-13 family (Rath and Kozielski, 2012). By regulating microtubule dynamics, KIF2C performs many functions in spindle assembly, sister chromatid separation, and error correction. However, in tumor cells, the regulation of KIF2C is disturbed and instead enhances mitotic defects and promotes tumor progression (Ritter et al., 2016).Previous studies have shown that KIF2C is overexpressed in some tumors and promotes their progression (Eichenlaub-Ritter, 2015). In cervical cancer (CC), KIF2C promotes its proliferation by inhibiting the P53 pathway (Yang et al., 2022). KIF2C is upregulated in endometrial cancer (EC), which can cause a malignant phenotype in EC and negatively correlates with CD8T cells, which have cancer suppressive effects (An et al., 2021). In hepatocellular carcinoma (HCC), KIF2C can promote epithelial-mesenchymal transition (EMT) for HCC progression (Mo et al., 2022). Through the TGF-1/Smad signaling pathway, KIF2C promotes thyroid cancer proliferation and migration (Lin et al., 2021). The above studies suggest that KIF2C acts as an oncogene and is associated with immune infiltration.Nevertheless, the mechanism of KIF2C in pan-cancer is poorly understood and needs to be studied systematically.

Tumor microenvironment influences tumorigenesis, proliferation and patient prognosis (Quail and Joyce, 2013).TME contains a variety of cell types such as tumor cells, immune cells, stromal cells, etc. Previous studies have shown that Tregs and TAMs in TME can impede anti-tumor immune function and thus promote tumor progression. (Petitprez et al., 2020). Soluble factors secreted by tumor or stromal cells can also induce the development of immune resistance, such as TGFβ, FGF2, and PDGF secreted by CAFs (Sadeghi Rad et al., 2021). Therefore, it is crucial to explore a new marker to improve the effectiveness of immunotherapy.

In this work, we investigated the prognosis of KIF2C in pan-cancer and its relationship with immune infiltration, and found that KIF2C was associated with cell cycle after enrichment analysis, and finally, we investigated the correlation between KIF2C expression and anticancer drug sensitivity. Our study demonstrates that KIF2C can serve as a prognostic marker in several tumors. They are correlated with immune infiltration and may be promising targets for enhancing the curative effect of immunotherapy.

## Materials and Methods

### The Cancer Genome Atlas

TCGA is a public platform containing oncogene information. The expression data, TMB data, MSI data and clinical data of TCGA for 33 tumor types can be downloaded through UCSC Xena online database (https://xenabrowser.net/) (Goldman et al., 2020). The abbreviations and meanings of the 33 tumor types are given in the Supplementary Table S1.

### UALCAN

UALCAN (http://ualcan.path.uab.edu) (Chen et al., 2019) is an online database that allows the analysis of gene expression data from TCGA. In the current study, we evaluated protein expression levels of KIF2C in LIHC using the “CPTAC analysis” module.

### Tumor Immune Estimation Resource 2.0

TIMER 2.0 (http://timer.cistrome.org/) (Li et al., 2017) is a database that allows the analysis of immune infiltration in different tumor types. In this study, the differential expression of KIF2C in tumor and normal tissues was performed by the “Gene_DE” module, the correlation of KIF2C expression with the level of immune infiltration in 33 tumor types was visualized by the “Gene” module, and the prognosis of KIF2C expression and immune cell infiltration was analyzed by the “Outcome” module. Finally, the “Correlation” module was used to analyze the relationship between KIF2C expression and immune marker subgroups in KIRC, LGG, LIHC and TYHM.

### Gene Enrichment Analysis

GO and KEGG enrichment analysis can be used as a way to explore gene function. In this study, we divided the expression of KIF2C into high and low groups according to the median and then performed enrichment analysis using clusterprofiler in R.

### Drug Sensitivity Analysis

CellMiner (Shankavaram et al., 2009; Reinhold et al., 2012)is a database that integrates molecular and pharmacological data from NCI-60 cancer cell lines. Data on drug sensitivity was obtained from CellMiner, *p* <0.05 was used as a screening condition and correlation analysis was performed by the spearman’s test. Computational Analysis of Resistance (CARE) (Jiang et al., 2018)is a database that can be used to calculate CARE scores to predict the correlation between target genes and the efficacy of targeted drugs.

### GeneMania

GeneMania is an online database that can predict the function of specific genes by building protein-protein interaction networks (Warde-Farley et al., 2010). The selected genes can be analyzed by bioinformatics methods. In this study, we constructed a PPI network for KIF2C through GeneMania to explore the function of KIF2C. In this study, we constructed a PPI network of KIF2C through GeneMania to explore the function of KIF2C.

### Immunohistochemistry (IHC)

To explore the protein expression level of KIF2C, we examined the expression level of KIF2C in paraffin-embedded tissues of liver cancer and normal liver tissues by IHC staining. Tissue sections were treated with anti-KIF2C primary antibody (1:200, Proteintech), and the protocol is described in (Zhang et al., 2021).

### Cell Culture and Quantitative Real-Time PCR

All cell lines in this study were obtained from the Oncology Laboratory of Tongji Hospital, Huazhong University of Science and Technology. Among them, BGC-823, SGC7901, and LO2 cell lines were cultured by RPMI-1640 complete medium, while MGC-803, Hep-3B, and MHCC97-H cell lines were cultured by DMEM complete medium at 37°C in 5% CO2.

Total RNA was extracted from various cell lines using TRIzol reagent (Takara, Shiga, Japan). The mRNA expression levels of KIF2C in each cell line were subsequently verified by RT-PCR according to the SYBR-Green (Takara, Shiga, Japan) ‘s protocol. The relative expression was calculated by 2-ΔΔC t and normalized using GAPDH as an internal reference.

### Statistical Analysis

We performed statistical analysis via R (version 4.1.0), forestplot based on the forestplot package for visualizing the hazard ratio (HR) of KIF2C in pan-cancer, survival analysis via the survminer and survivor packages, box plots and scatter plots via the ggplot2 package, and reshape2 package to plot correlation heat map. ROC curves are plotted via the ROC package, and radar plots are visualized via the ggradar package. The correlation of KIF2C expression with TMB, MSI, MRR immune checkpoint genes and drug sensitivity was analyzed by the spearman test, and the analysis of the difference in pan-cancer expression was analyzed by Wilcox test. *p* <0.05 was accepted as statistically significant.

## Results

### KIF2C is Overexpressed in Multiple Tumors

**FIGURE 1:**
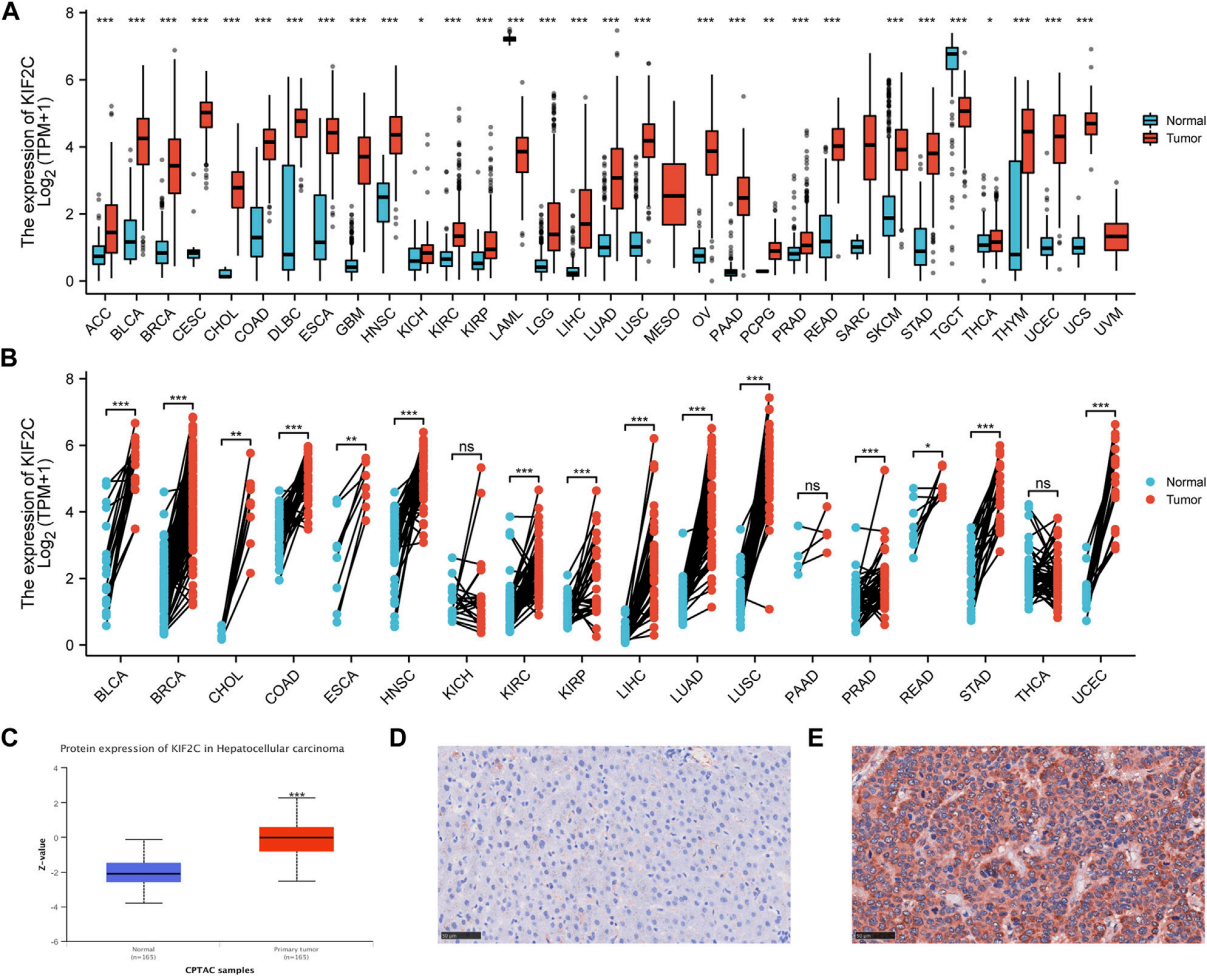
Pan-cancer KIF2C expression analysis **(A)** KIF2C expression levels in pan-cancer from TGCA and GTEx **(B)** Expression levels of KIF2C in paired samples **(C)** KIF2C protein level in hepatocellular carcinoma was examined using the CPTAC dataset. **(D)** Low expression of KIF2C in normal tissues. **(E)** High expression of KIF2C in liver cancer tissues. *p < 0.05, **p <0.01, ***p <0.001.

We analyzed the mRNA levels of KIF2C in 33 tumor types by TIMER 2.0 database and GEPIA database and found KIF2C was highly expressed in BLCA, BRCA, CESC, CHOL, COAD, DLBC, ESCA, GBM, HNSC, KIRC, KIRP,LGG, LIHC, LUAD, LUSC, OV, PCPG, PRAD, READ, SKCM, STAD, THYM, UCEC, and UCS (Figures 1A,B). Moreover, the protein level of KIF2C was analyzed by UALCAN database and found that KIF2C was significantly overexpressed in BRCA, KIRC, COAD, OV, LUAD, and LIHC (Figure 1C). In addition, we verified the mRNA expression levels of KIF2C, and we found that KIF2C expression was significantly higher in human HCC cell lines (MHCC97-H, Hep-3B) than in normal human hepatocytes (LO2) (Supplementary Figure S1A). However, there was no significant difference in the expression of KIF2C in gastric cancer cell lines (Supplementary Figure S1B). We analyzed the mRNA levels of KIF2C in 33 tumor types by the TCGA database and GTEx database, and combined with differential analysis of paired samples, we found that KIF2C was highly expressed in ACC, BLCA, BRCA, CHOL, COAD, DLBC, ESCA, HNSC, KIRC, KIRP, LGG, LIHC, LUAD, LUSC, OV, PCPG, PRAD, READ, SKCM, STAD, THYM, UCEC, and UCS (Figures 1A,B). In addition, analysis of KIF2C protein levels by the UALCAN database revealed that KIF2C was overexpressed in LIHC (Figure 1C). We verified the expression level of KIF2C in liver cancer tissues by immunohistochemistry, and the results were consistent with the database (Figures 1D,E). In addition, we verified the mRNA expression levels of KIF2C, and we found that KIF2C expression was significantly higher in human HCC cell lines (MHCC97-H, Hep-3B) than in normal human hepatocyte lines (LO2) (Supplementary Figure S1A). However, there was no significant difference in the expression of KIF2C in gastric cancer cell lines (Supplementary Figure S1B).

### KIF2C is a Potential Prognostic Marker for Multiple Tumors

**FIGURE 2:**
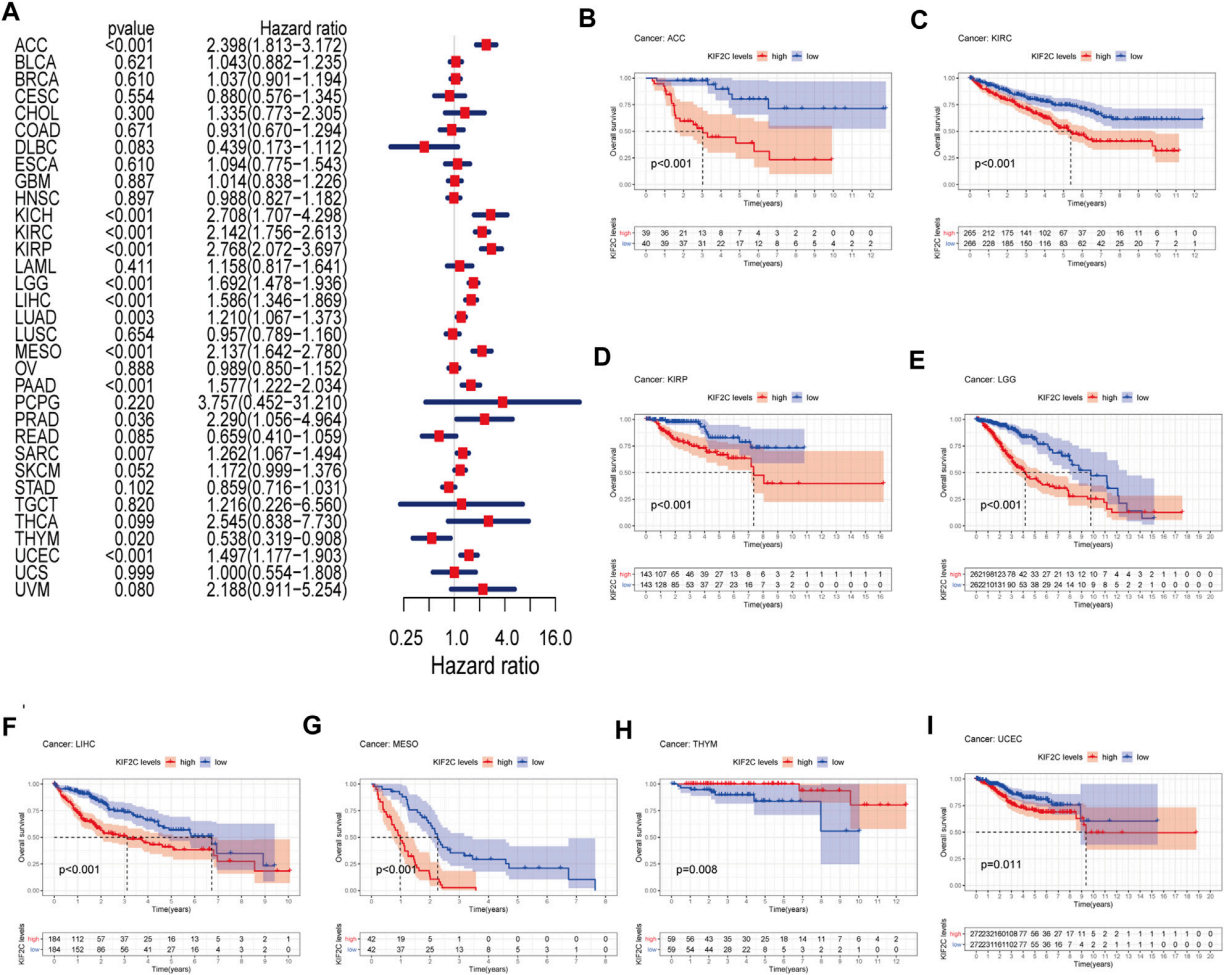
OS analysis of KIF2C in pan-cancer **(A)** Forest plot shows hazard ratios of KIF2C in pan-cancer. **(B–I)** Kaplan-Meier survival curves of pan-cancer with high and low KIF2C expression analysed by the TCGA database.

We divided the tumors into two groups, high and low expression, according to the median expression of KIF2C in various tumor types. We investigated the prognostic value of KIF2C in TCGA pan-cancer by univariate Cox regression analysis. The results are presented in the form of a forest plot (Figure 2A). KIF2C overexpression could significantly impact ACC (HR = 2.398), KICH (HR = 2.708), KIRC (HR = 2.142), KIRP (HR = 2.768), LGG (HR = 1.692), LIHC (HR = 1.586), LUAD (HR = 1.210), MESO (HR = 2.137), PAAD (HR = 1.577), PRAD (HR = 2.290), SARC (HR = 1.262), THYM (HR = 0.538), and UCEC (HR = 1.497) for overall survival. KIF2C upregulation related to poorer OS prognosis of the above tumors, except for TYHM. Except for TYHM, KIFU2C upregulation is associated with a poorer OS prognosis.

Subsequently, we performed Kaplan-Meier survival analysis and found that KIF2C upregulation was significantly associated with poorer prognosis in ACC, KIRC, KIRP, LGG, LIHC, MESO, and UCEC, while it was significantly associated with better prognosis in THYM (Figure 2B).

In addition, we also investigated the correlation of KIF2C with PFI, DFI and DSS, and the results showed that KIF2C was significantly associated with poor prognosis in several cancer types (Supplementary Figure S2). The results of OS, PFI, DFI and DSS survival analysis consistently indicated that KIF2C was a risk factor for ACC,KIRP, LIHC,LUAD, PRAD, SARC, and UCEC.

### Correlation of KIF2C Expression With TMB, MSI and DNA Mismatch Repair (MMR) in Multiple Tumors

**FIGURE 3:**
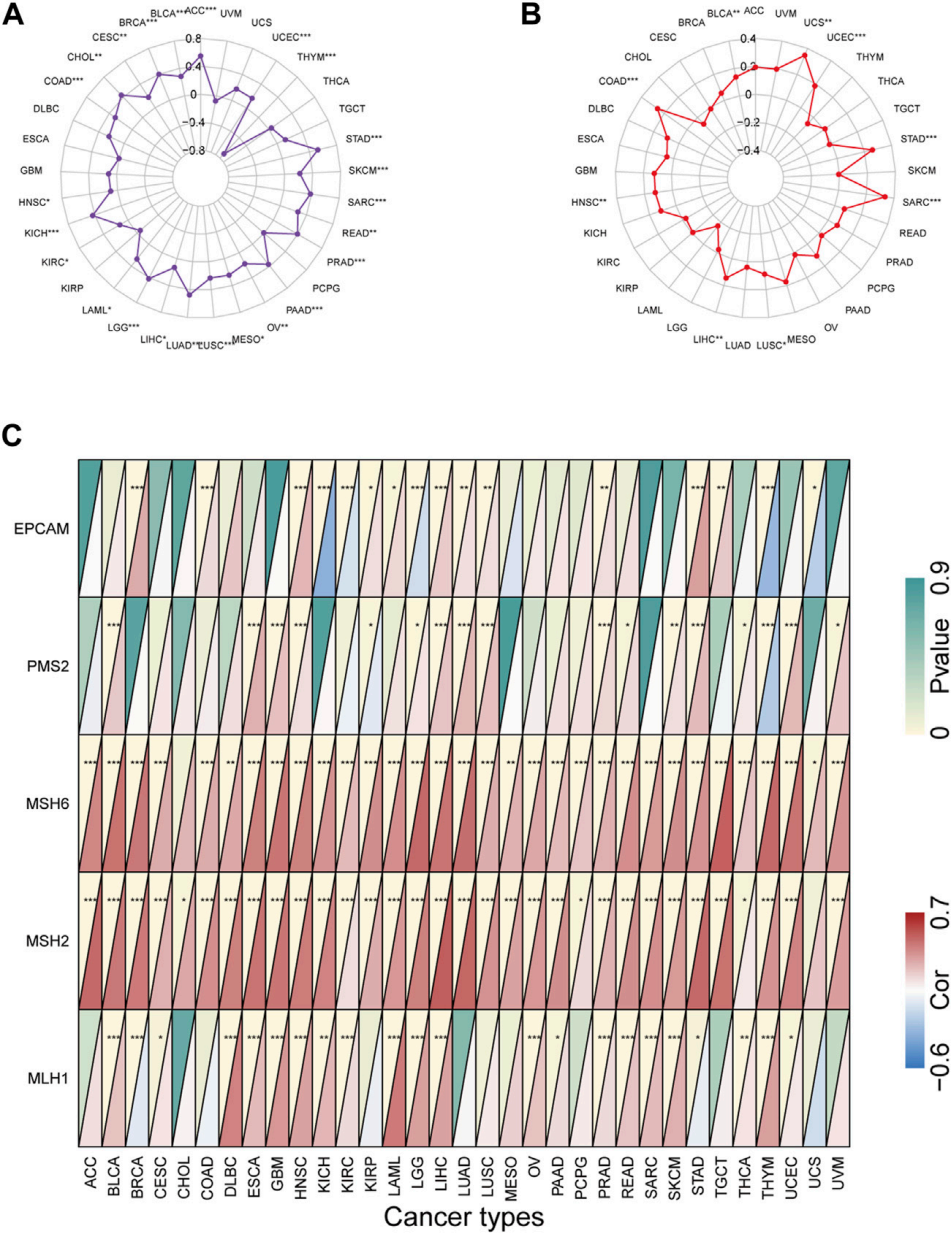
Correlation of KIF2C expression with tumor mutation burden (TMB), microsatellite instability (MSI), and mismatch repairs (MMRs) in pan-cancer **(A)**Radar map shows correlation of KIF2C expression with TMB in pan-cancer. **(B)** Radar map shows correlation of KIF2C expression with MSI in pan-cancer. **(C)** Heatmap shows correlation of KIF2C expression with 5 MMRs related genes in pan-cancer.

Previous studies have shown that TMB and MSI-H are predictive biomarkers for tumor immunotherapy TMB and MSI-H have previously been demonstrated to be predictive biomarkers for tumor immunotherapy (Rizzo et al., 2021), thus we analyzed the correlation between KIF2C expression and different tumor TMB, MSI and MMRs. Figure 3A shows that high KIF2C expression was significantly correlated with TMB of 24 tumors, and only significantly negatively correlated with THYM. Figure 3B shows that high expression of KIF2C was positively correlated with MSI in BLCA, COAD, HNSC, LIHC, LUSC, SARC, STAD, UCEC, and UCS. dMMR can induce MSI production, so we evaluated the association between the expression level of KIF2C and the mutation levels of five MMR genes (MLH1, MSH2, MSH6, PMS2, and EPCAM). Figure 3C shows that KIF2C was significantly associated with MMR genes in 30 tumors.

### Epigenetic Analysis of KIF2C

**FIGURE 4:**
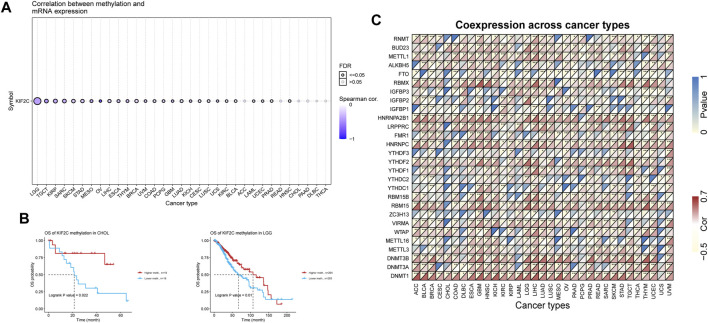
Epigenetic analysis of KIF2C. **(A)** Correlation of KIF2C methylation with mRNA expression. **(B)** Prognostic analysis of KIF2C methylation in CHOL and LGG.**(C)** Heatmap of the correlation between KIF2C and transmethylase genes, m6A-related genes and m7G-related genes in pan-cancer.

We performed methylation analysis of KIF2C through the GSCA database (Liu et al., 2018) and found that KIF2C was significantly negatively correlated with KIF2C methylation levels in most tumors (Figure 4A), and KIF2C hypomethylation was associated with poor prognosis of LGG and CHOL by prognostic analysis (Figure 4B). We further investigated the correlation between KIF2C and DNA methyltransferase (DNMT)-related genes, N6-methyladenosine (m6A) and 7-methylguanosine (m7G)-related genes, and the results showed that KIF2C was significantly correlated with the above-mentioned related genes in a variety of tumors (Figure 4C), suggesting that KIF2C is closely related to epigenetic modifications that affect tumor progression.

### Correlation Between KIF2C Expression and Tumor Microenvironment of Multiple Tumors

**FIGURE 5:**
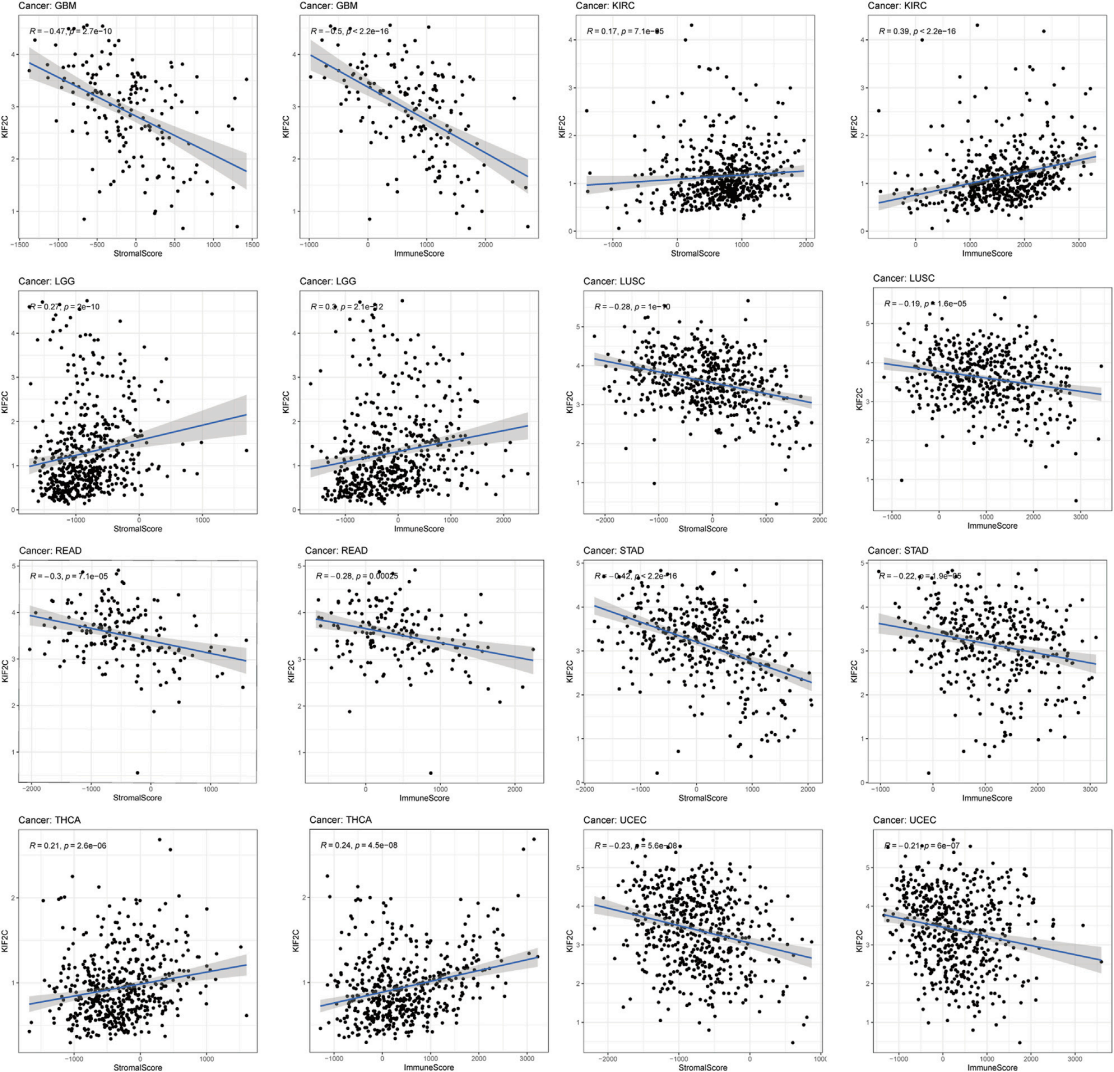
The correlations between KIF2C expression and the stromal and immune cell’s level of tumor microenvironments in pan-cancer.

TME plays an important role in promoting tumor progression and consists mainly of immune cells and stromal cells (Binnewies et al., 2018). We obtained immune scores and stromal scores of 33 tumors from TCGA database by the ESTIMATE algorithm and performed correlation analysis. Figure 5 showed that KIF2C expression was significantly positively correlated with immune and stromal scores in KIRC, LGG, and THCA, but negatively correlated with those in GBM, LUSC, STAD, and UCEC. In addition, high KIF2C expression was negatively correlated with stromal scores in BRCA, COAD, HNSC, LIHC, LUAD, SARC, SKCM, TGCT, and THYM, and negatively correlated with immune scores in DLBC (Supplementary Figure S3).

### Immune Infiltration Analysis of KIF2C

**FIGURE 6:**
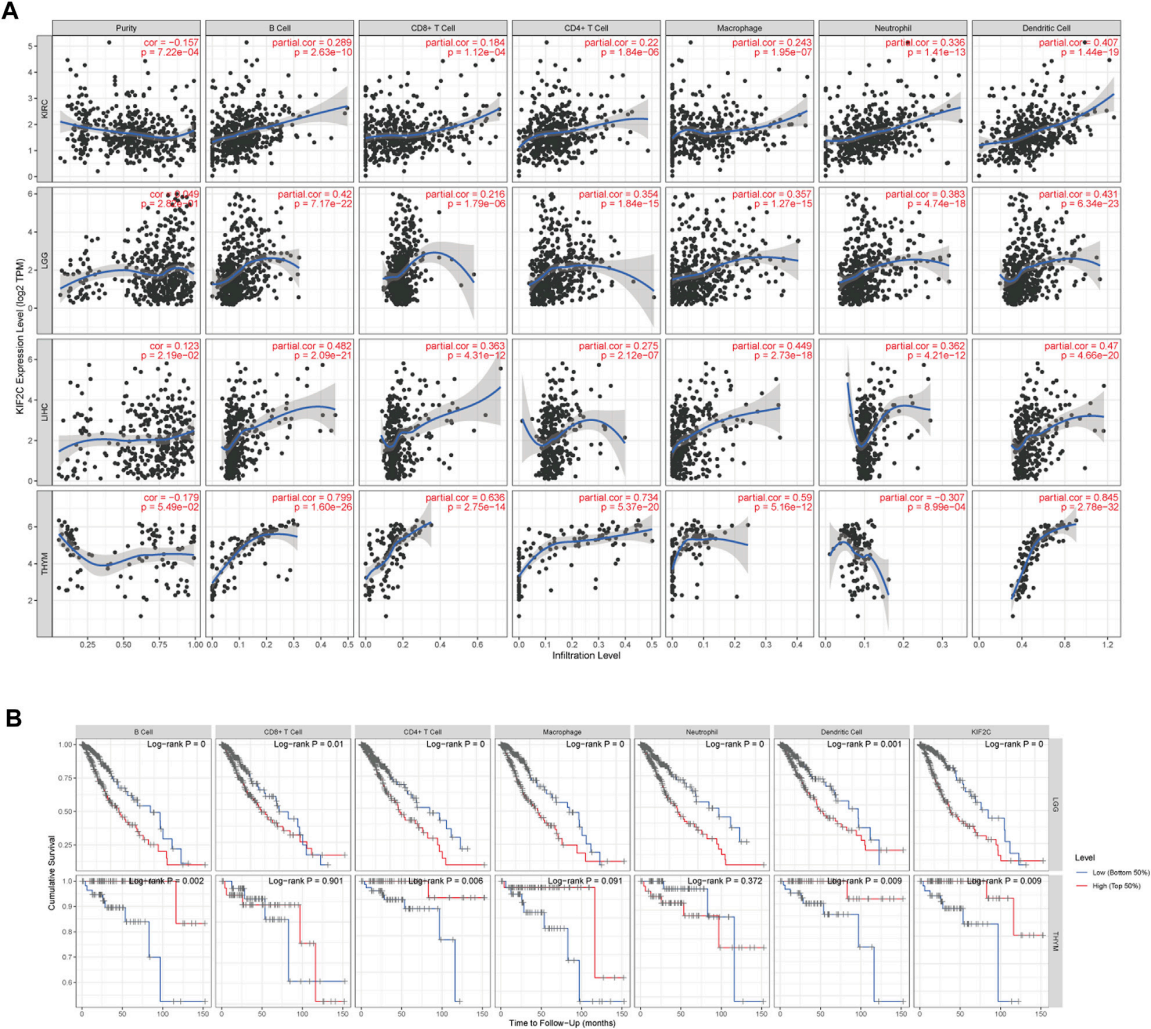
**(A)** Correlation of KIF2C expression with immune infiltration level in KIRC, LGG, LIHC and THYM. **(B)** KM analysis of immune cells infiltration levels and expression levels of KIF2C in LGG and THYM.

Using the TIMER database, we examined the correlation between KIF2C expression and immune infiltration levels in various cancers. In Figure 6A, KIF2C expression in KIRC, LGG, LIHC, and TYHM was significantly correlated with B cell, CD8^+^ T cell, CD4^+^ T cell, macrophage cell, neutrophil cell, and dendritic cell. Except for the negative correlation between KIF2C and the neutrophil cell of THYM, all of them were significantly positively correlated. By KM analysis (Figure 6B), high KIF2C expression and high infiltration levels of the six immune cells were significantly correlated with poor OS in LGG, whereas high KIF2C expression and high infiltration levels of B cells, CD4^+^ T cells, and dendritic cells were correlated with better OS in THYM.

**FIGURE 7:**
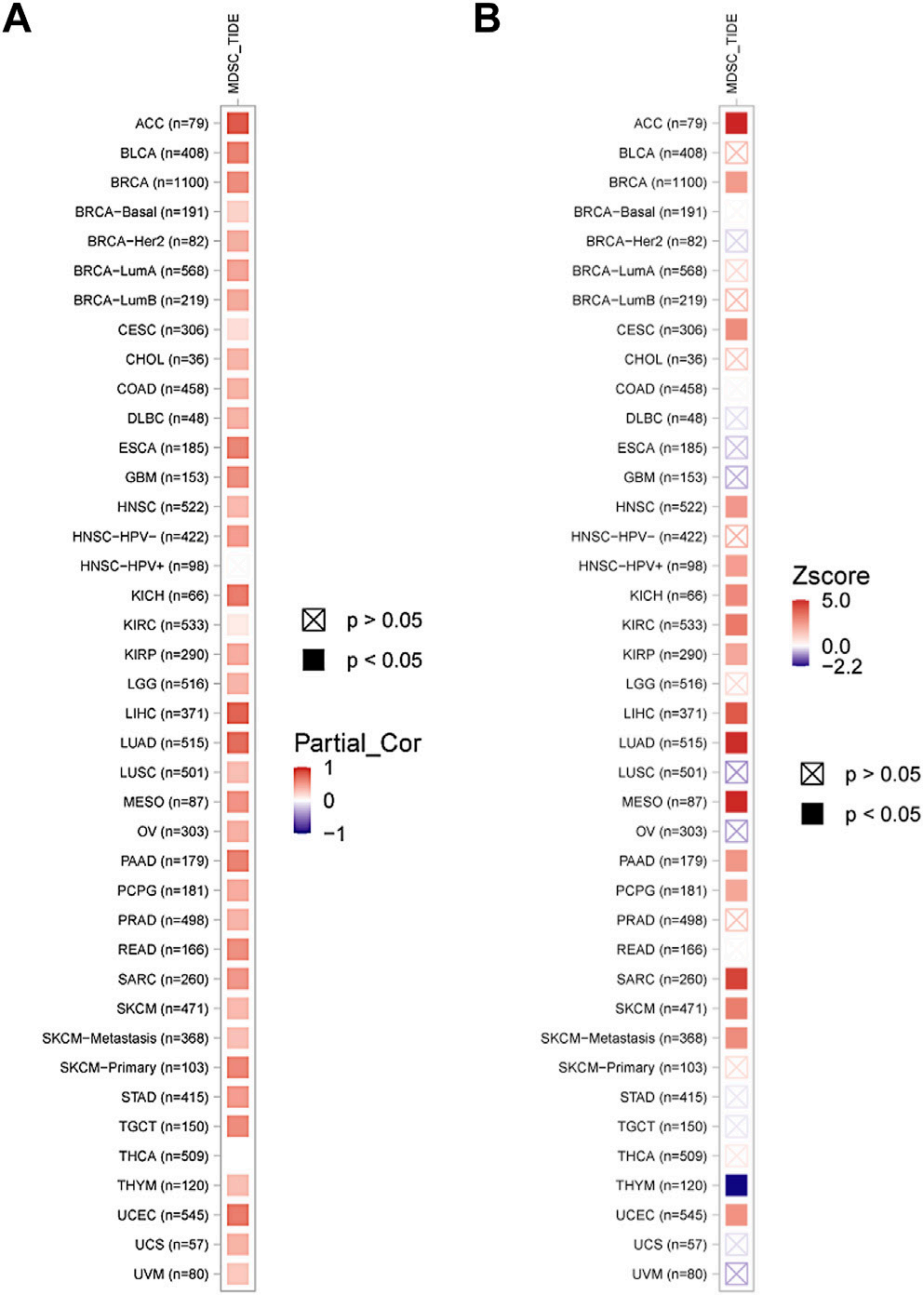
**(A)** Correlation of KIF2C with MDSCs in pan-cancer. **(B)** The prognosis analysis of MDSCs, red means poor prognosis, blue means good prognosis.

Myeloid-derived suppressor cells (MDSCs) have immunosuppressive characteristics, and we analyzed the correlation between KIF2C and MDSCs in pan-cancer by TIMER 2.0 and found that KIF2C was significantly positively correlated with MDSCs in most tumors (Figure 7A). Moreover, MDSCs was associated with a poorer prognosis (Figure 7B).

**FIGURE 8:**
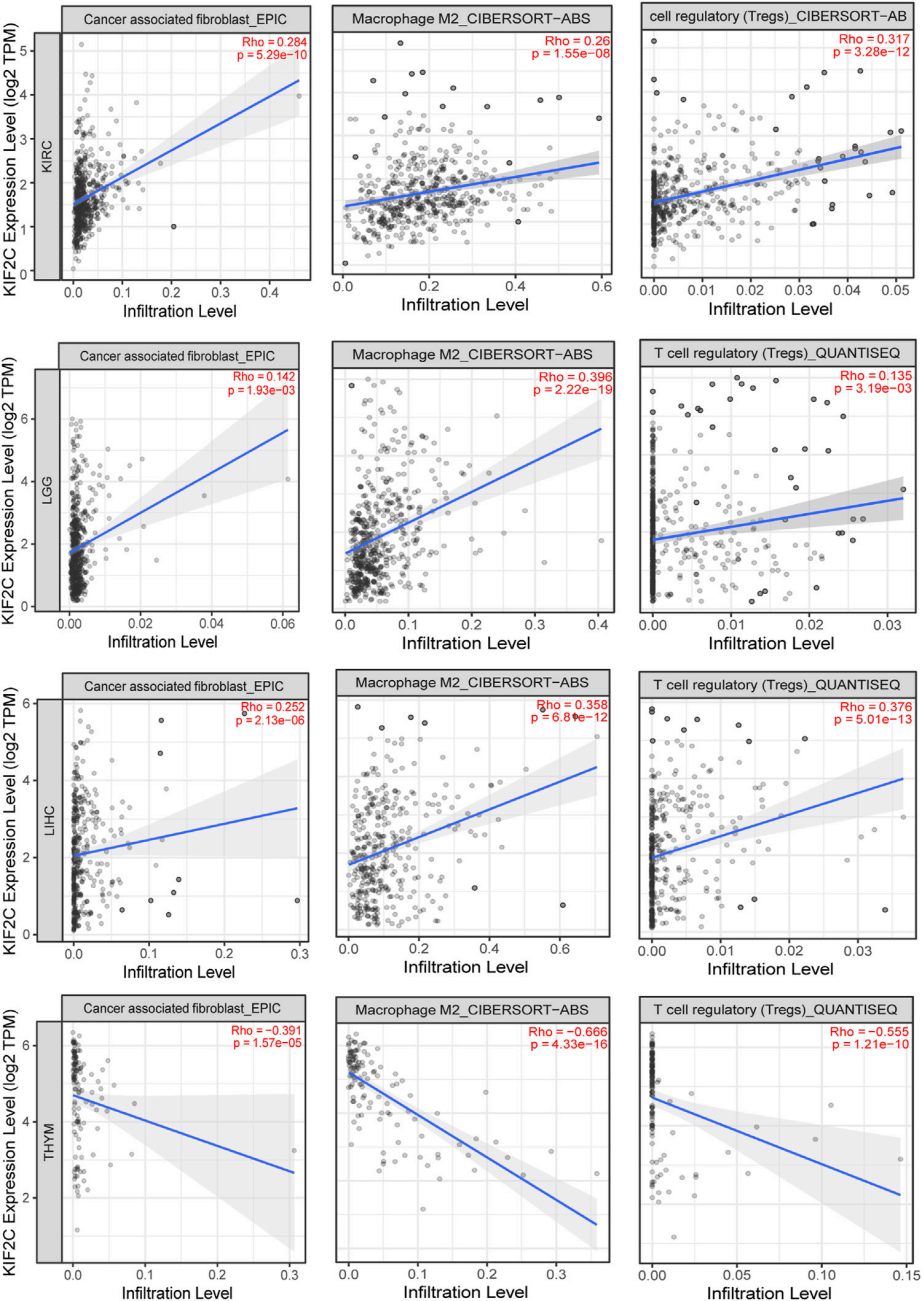
Correlation of KIF12C expression with CAFs, M2 macrophages and Tregs in KIRC, LGG, LIHC, and THYM.

Additionally, we used EPIC, CIBERSORT-ABS, and QUANTISEQ algorithms to analyze the correlation of KIF2C with cancer-associated fibroblasts, M2 macrophages, and Tregs in KIRC, LGG, LIHC, and THYM. We found that KIF2C was positively correlated with CAFS, M2 macrophages, and Tregs in KIRC, LGG, and LIHC. However, in THYM, KIF2C was negatively correlated with all three of these cells (Figure 8). We then analyzed the association between KIF2C expression and immune markers in KIRC, LGG, LIHC, and TYHM, and to reduce bias, we corrected the data according to tumor purity. Supplementary Table S2 shows that KIF2C expression in KIRC was mainly positively correlated with TAM, M2 macrophages, Tregs, and T cell exhaustion. In contrast, in TYHM, KIF2C expression was negatively correlated mainly with M2 macrophages, monocytes, T cell exhaustion, and TAM. It is evident that KIF2C has an important role in immune infiltration.

### Correlation of KIF2C Expression With immune Checkpoint Genes and immune Response in Multiple Tumors

To explore the relationship between KIF2C and immunotherapy, we performed a pan-cancer correlation analysis of KIF2C with 47 immune checkpoint (ICP) genes. Supplementary Figure S4 shows that KIF2C expression correlated with 42 genes in THCA, 39 genes in PRAD and LIHC, and 38 genes in BRCA and THYM. More than 30 ICP genes were associated in with KIRC, UCEC, LGG, and GBM.KIF2C was positively correlated with ICP genes in BLCA, BRAC, HNSC, KICH, KIRC, KIRP, LGG, LIHC, MESO, PAAD, and THCA, suggesting that KIF2C may exerts exert an immunosuppressive effect in these tumors and that targeting KIF2C can achieve better immunotherapy outcomes. However, KIF2C is mainly negatively correlated with ICP genes in COAD, DLBC, GBM, LUAD, LUSC, READ, SKCM, STAD, THYM, and UCEC, which means that targeting KIF2C in such tumors may not achieve favorable immunotherapy outcomes.

**FIGURE 9:**
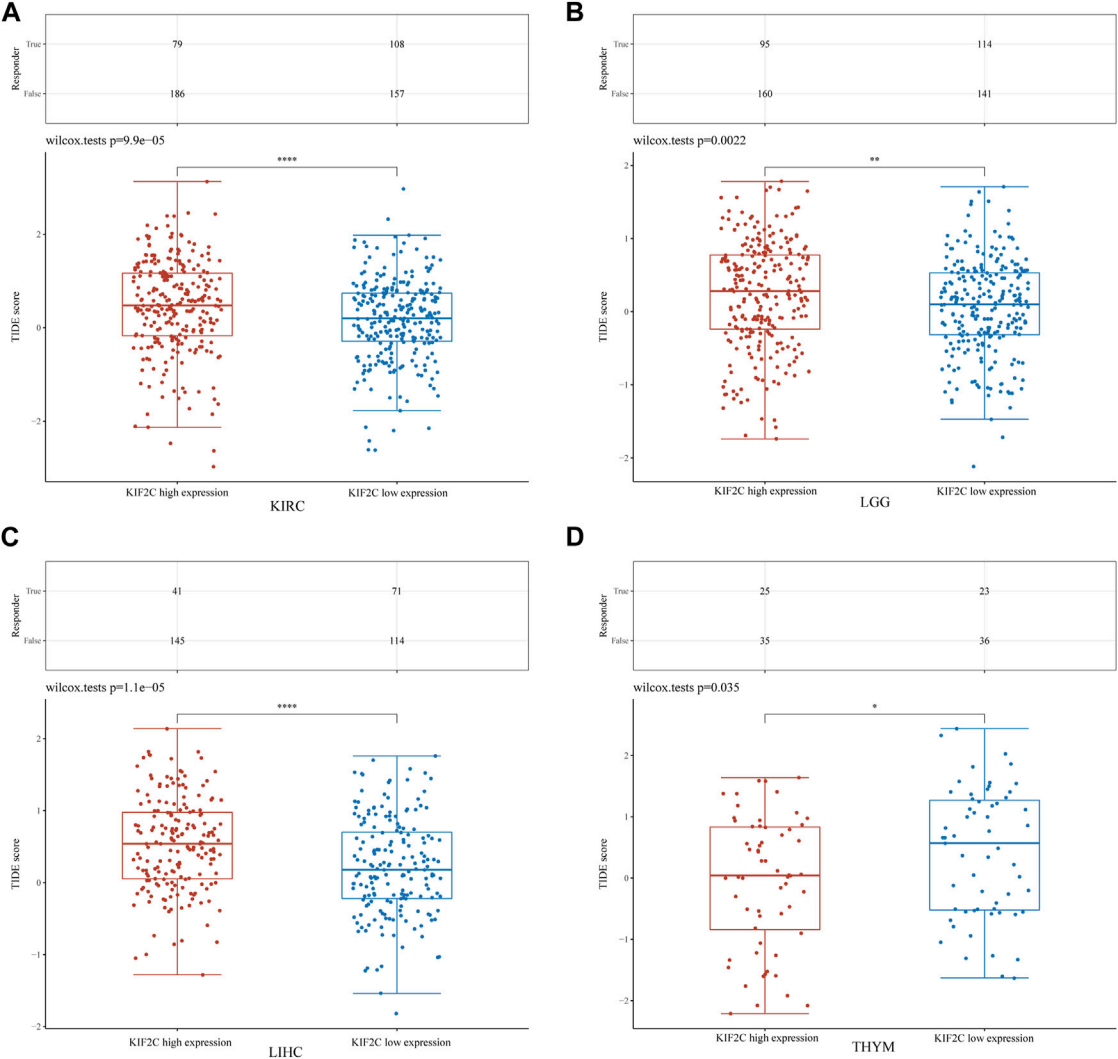
**(A–D)** TIDE score of KIF2C high and low expression groups in KIRC, LGG, LIHC, and THYM.

To explore the KIF2C expression effect on immune checkpoint blockade (ICB) treatment response more deeply, we used the TIDE algorithm to obtain the TIDE scores of KIF2C in KIRC, LGG, LIHC and THYM. High TIDE scores were associated with poor response to ICB therapy and short survival after receiving ICB therapy (Jiang et al., 2018). We found that the KIF2C high expression group had higher TIDE scores in KIRC, LGG and LIHC, while it had lower TIDE scores in THYM (Figures 9A–D), suggesting that KIF2C may promote immune escape and cause poor ICB response in KIRC, LGG and LIHC, but in THYM the opposite is true. This is consistent with our previous findings.

### GO and KEGG Analysis of KIF2C Expression

**FIGURE 10:**
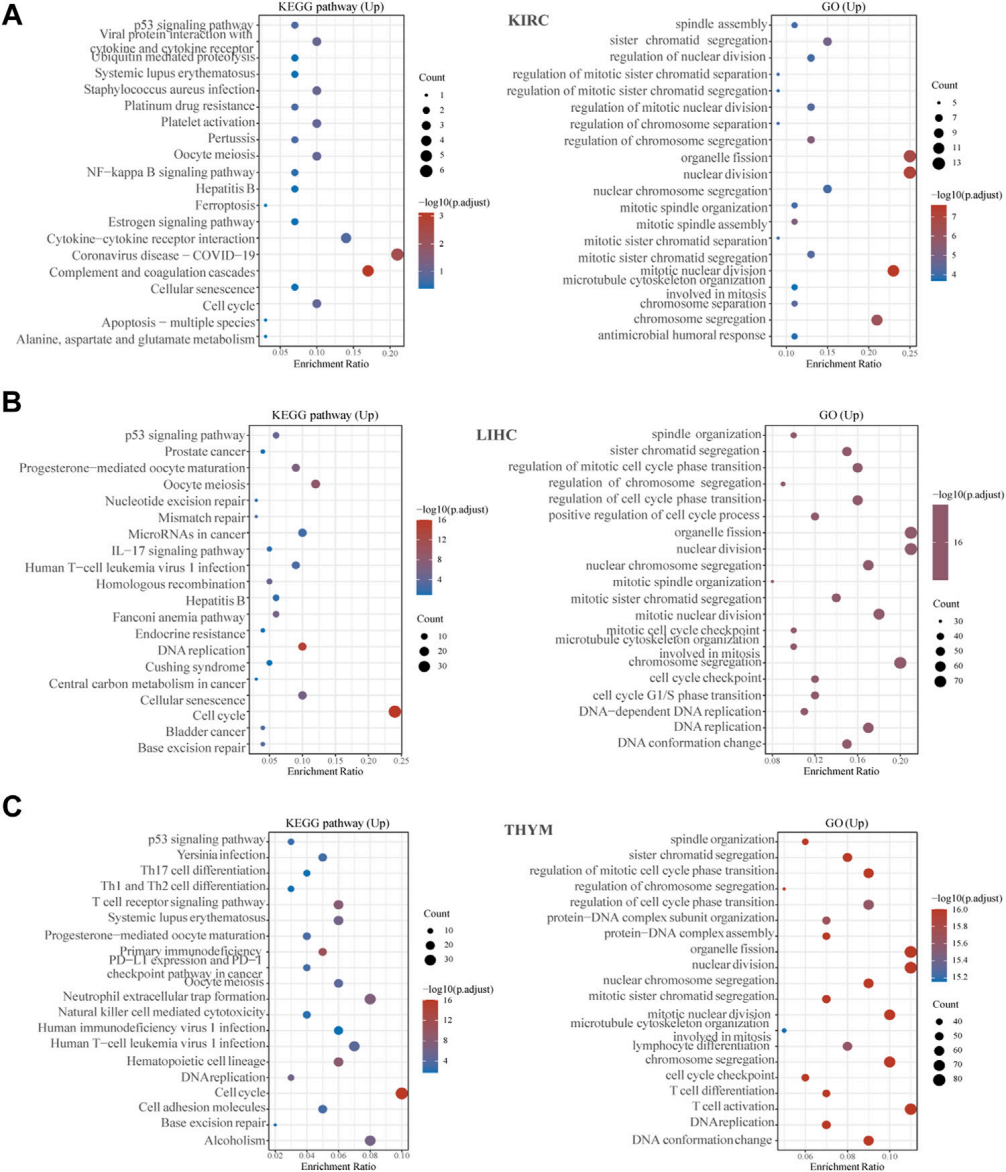
Gene enrichment analysis of KIF2C in KIRC, LGG, and THYM. **(A–C)** Top 20 GO and KEGG pathway analysis in the specified tumor types.

To explore the biological role of KIF2C in tumors, we performed GO and KEGG analysis on three tumors, KIRC, LIHC, and THYM. As shown in Figure 10, we found that KIF2C is involved in the regulation of the cell cycle in all three tumors and is related to immune regulation. In KIRC, KIF2C is involved in immune-related processes such as “complement and coagulation cascade,” “cytokine-cytokine receptor interaction,” and also, KIF2C is associated with “Coronavirus disease-COVID-19”. In LIHC, KIF2C is correlated with the “IL-17 signaling pathway”. In THYM, KIF2C was correlated with “T cell receptor signaling pathway”and “T cell activation”. It can be seen that KIF2C is closely related to immunity but plays a different immune role depending on the type of cancer.

### PPI Network of KIF2C Analysis

**FIGURE 11:**
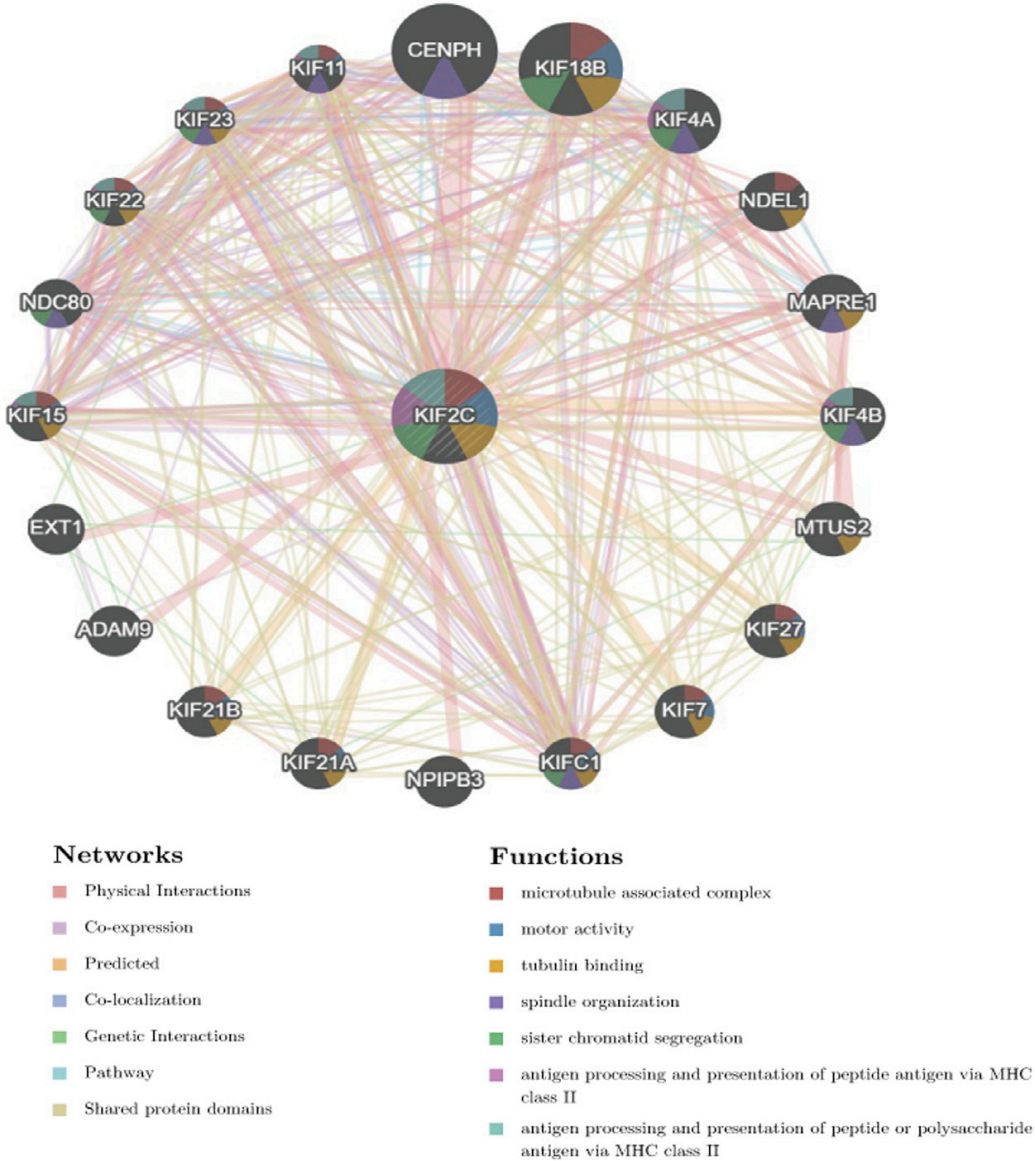
PPI network of KIF2C constructed using the GeneMania database.

We constructed the PPI network of KIF2C through the GeneMania database. Figure 11 shows that KIF2C interacts with genes such as CENPH, ADAM9, NDEL1, and EXT1 and is associated with biological functions such as tubulin binding, sister chromatid segregation, and MHC II antigen presentation, suggesting that KIF2C is involved in cell cycle and immune regulation. In addition, previous studies found that the KIF2C-related CENPH, EXT1, can promote tumor progression (Wu et al., 2015; Lu et al., 2017; Kong et al., 2021) and ADAM9 has a significant role in tumor growth, metastasis, and immune evasion (Chou et al., 2020).

### Drug Sensitivity Analysis of KIF2C

**FIGURE 12:**
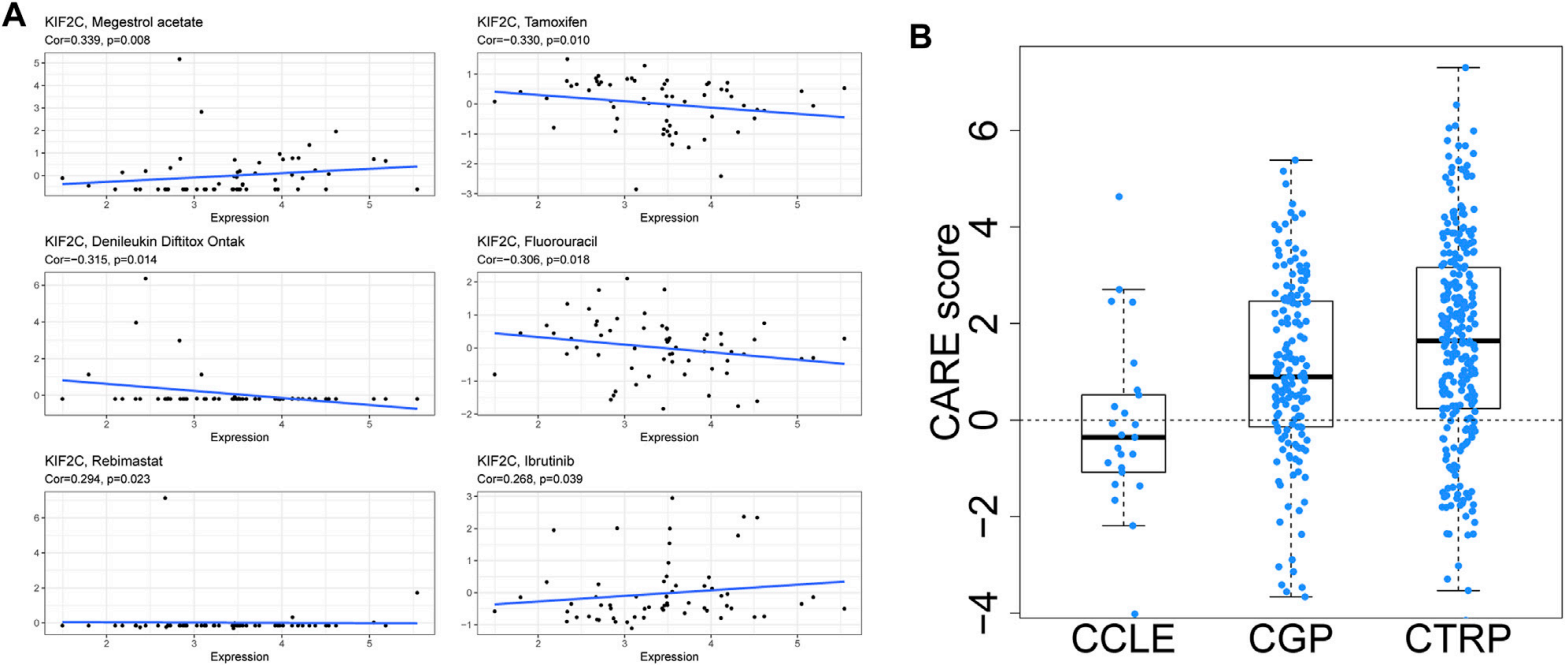
Anticancer drug sensitivity analysis of KIF2C. (A) CellMiner's drug sensitivity analysis. (B) CARE analysis

Using the CellMiner database, we looked at the relationship between KIF2C expression and drug sensitivity. As shown in Figure 12A, KIF2C expression was significantly positively related to the sensitivity of Megestrol acetate, Rebimastat, and Ibrutinib. In contrast, the sensitivity of Tamoxifen, Denileukin Diftitox Ontak and Fluorouracil was significantly negatively correlated. To further investigate, we analyzed the correlation between KIF2C expression and drug efficacy through the CARE database. The results showed that KIF2C expression was positively correlated with the sensitivity of PI3K-AKT signaling pathway inhibitors (MK-2206, Rigosertib), CDK1 inhibitor (Flavopiridol) and PLK1 inhibitor (BI2536), while it was correlated with the resistance to EGFR inhibitors (Figure 12B). Detailed information is presented in Supplementary Table S3.

### Construction of Nomogram

**FIGURE 13:**
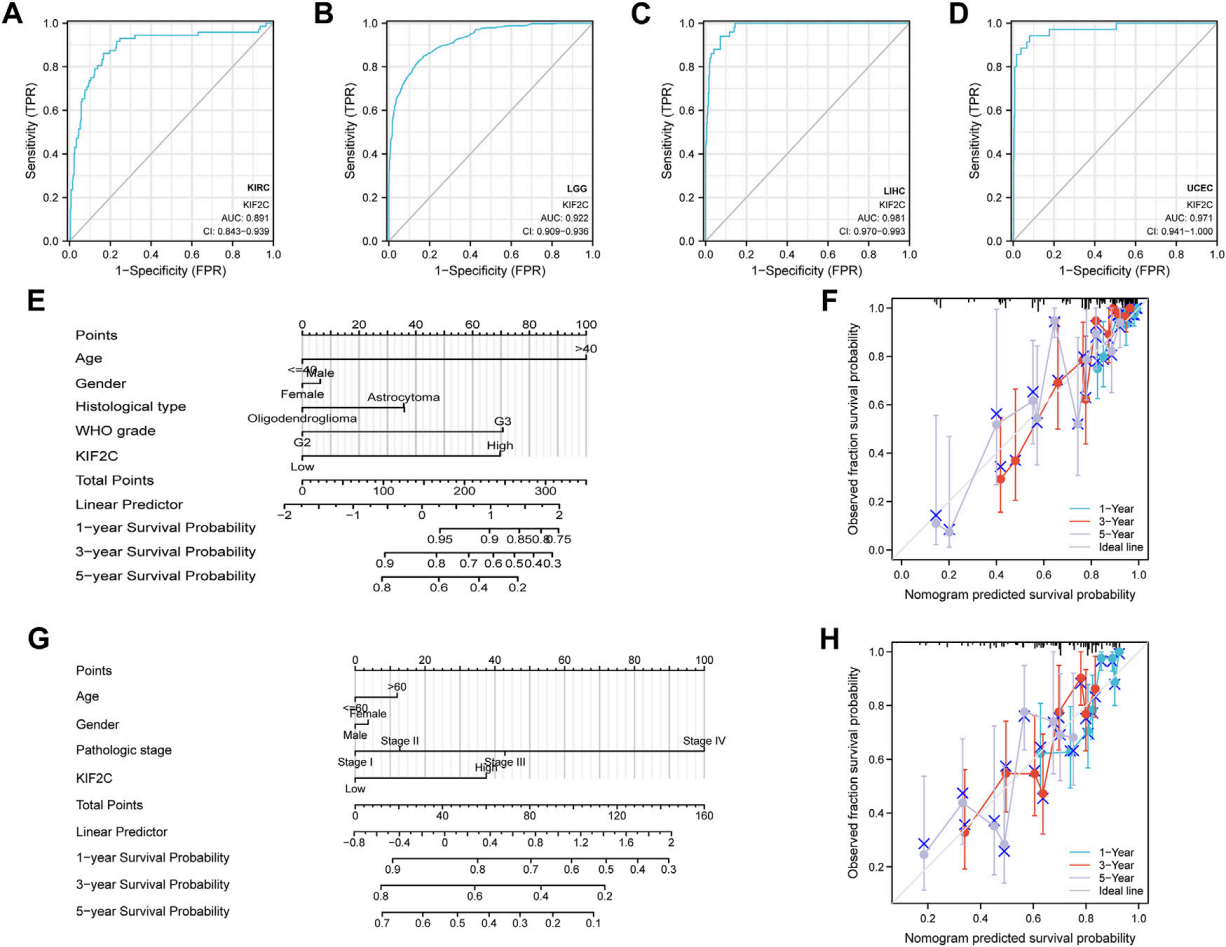
Prediction evaluation and construction of nomograms **(A–D)** ROC analysis of KIRC, LGG, LIHC, and UCEC by KIF2C. **(E)** Construction of nomograms for LGG **(F)** Calibration curve validation of LGG. **(G)** Nomogram construction for LIHC. **(H)** Calibration curve validation of LIHC.

We verified the predictive efficacy of KIF2C in tumors by ROC predictive effect on the prognosis of the above cancer types. In order to predict the prognosis of patients more accurately, we constructed a nomogram based on KIF2C for LGG and LIHC to predict the 1,3,5-year survival rate of patients and validated it with the calibration curves(Figures 13E–H). We found that the nomogram was in good agreement with the actual situation.

## Discussion

The function of KIF2C in cell division requires complex regulation by various kinases and phosphatases, such as Aurora A/B, Plk1, and Cdk1 (Sanhaji et al., 2011). However, when KIF2C is deregulated, it causes abnormal spindle formation and failed chromosome segregation, resulting in chromosomal instability and tumorigenesis (Sanhaji et al., 2011). Previous studies have identified that KIF2C was overexpressed in some cancers (Nakamura et al., 2007; Shimo et al., 2007; Ishikawa et al., 2008; Wei et al., 2020) and facilitates the growth and invasion of tumor cells. Currently, the function of KIF2C in tumors is not clear, and a comprehensive pan-cancer analysis is needed.

In this study, the mRNA levels of KIF2C were highly expressed in 28 tumors by TCGA database analysis. In addition, analysis of the UALCAN database revealed that the protein levels of KIF2C were significantly higher in HCC tumors than in adjacent tissues. The prognostic analysis revealed that KIF2C was negatively correlated with the OS of ACC, KIRC, KIRP, LGG, LIHC, MESO, and UCEC, and positively correlated with the OS of THYM only, which, combined with the results of Cox regression analysis, can be considered as a prognostic marker in several cancers.

Epigenetic alterations have important effects on tumor progression and immunosuppression, of which DNA methylation is one of the most important epigenetic regulators (Dawson and Kouzarides, 2012; Jones et al., 2019). We found that the mRNA levels of KIF2C were significantly correlated with methylation in different cancer types, and there was a correlation between DNMT and KIF2C expression, suggesting that DNA methylation may also be involved in the regulation of KIF2C. In LGG and CHOL, hypomethylation of KIF2C was significantly associated with poor prognosis. The mechanism may be related to the hypomethylation of KIF2C leading to tumorigenesis and overexpression of immunosuppression-related genes.

In addition to DNA methylation, internal modifications of RNA are also important and play an important role in tumor progression, especially m6A modification and m7G modification (Barbieri and Kouzarides, 2020). m6A modification can affect immune cell infiltration in the tumor microenvironment (Zhang et al., 2020), while m7G modification can cause immune escape (Devarkar et al., 2016). Therefore, we also explored the correlation between KIF2C and m6A and m7G-related genes, and found that KIF2C was significantly associated with m6A and m7G-related genes in most tumors, suggesting that the above RNA modifications may be involved in the regulation of KIF2C and thus influence the tumor progression.

Immunotherapy is emerging as an important modality in the treatment of cancer, such as ICB targeting CTLA-4 and PD-1 and its ligand (PD-L1), which can be used to treat tumors by reactivating anti-cancer immune responses (Petitprez et al., 2020). However, it was found that the interaction between the tumor cells in the TME and their surrounding stroma could affect the therapeutic effect of ICB and even cause drug resistance. Hence, the correlation between KIF2C and TME was explored. Analysis by the ESTIMATE algorithm revealed that KIF2C expression was significantly positively correlated with immune and stromal scores in KIRC, LGG, and THCA, and significantly negatively correlated in GBM, LUSC, STAD, and UCEC, suggesting a possible role of KIF2C expression in tumor immunity and TME. We analyzed the relationship between KIF2C expression and immune cell infiltration through the TIMER database and found that KIF2C expression was significantly correlated with all six immune cell types in KIRC, LGG, LIHC, and TYHM, and by correlation with immune markers, we discovered that KIF2C expression was positively correlated with CAFs, TAMs, M2 macrophages, Tregs, and MDSCs. TAMs are usually dominated by the M2 subtype. Unlike the M1 subtype, which inhibits tumor progression, the M2 subtype can promote tumor cell invasion, tumor cell invasion and metastasis, and the generation of immune tolerance (Shu and Cheng, 2020).whereas Tregs can lead to T cell exhaustion and are contributes to tumor evasion from immune surveillance and is a major obstacle to immunotherapy While Tregs can cause T cell exhaustion and contribute to tumor evasion from immune surveillance, which is a major barrier to immunotherapy (Wolf et al., 2015), MDSCs is a group of myeloid cells that arise under pathological conditions of cancer, MDSCs can promote tumor progression as well as tumor immunosuppression MDSCs are a group of myeloid cells that arise under pathological conditions of cancer and can promote tumor progression as well as tumor immunosuppression (Gabrilovich, 2017), CAFs are important components of TME and can promote tumor invasion, metastasis, and chemoresistance. In addition, CAFs can promote the recruitment, activation, and immunosuppressive behavior of immunosuppressive cells, including M2-type TAMs, Tregs and MDSCs (Mao et al., 2021). The above indicates that KIF2C expression is associated with immunosuppression.

In THYM, KIF2C strongly correlated with CD8^+^ T cells with tumor suppressive effects and negatively correlated with CAFs, M2 macrophages, Tregs, and TIDE score, suggesting that KIF2C expression in THYM is a protective factor that predicts a better ICB response. In contrast, in KIRC, LGG, and LIHC, KIF2C was positively correlated with CAFs, M2 macrophages, Tregs, MDSCs, and TIDE scores, suggesting that KIF2C high expression in these three tumors is associated with immunosuppression and poorer immunotherapeutic response, and thus KIF2C is a prognostic risk factor for KIRC, LGG, and LIHC, and targeting KIF2C may improve immunotherapeutic outcomes.

Previous studies have shown that defective MMR leads to MSI-H (Lu et al., 2021), and MSI and TMB are considered to be predictable markers predictors of ICB response (Chang et al., 2018; Sha et al., 2020), so the correlation of KIF2C with MMRs, MSI, and TMB was explored and it was found that KIF2C expression was significantly correlated with TMB in 24 tumors, MSI in 9 tumors, and MMRs in 30 tumors. We also analyzed the relationship between KIF2C and 47 ICP genes and found that KIF2C expression was significantly associated with several ICP genes, suggesting that KIF2C can be considered as a biomarker to predicting predict the response of to immunotherapy. We analyzed the biological functions of KIF2C by gene enrichment analysis. We found that in addition to the cell cycle, KIF2C expression was also significantly associated with complement and coagulation cascade, IL-17 signaling pathway, and cytokine-cytokine receptor interaction, suggesting that KIF2C is involved in tumor regulation of immunity.

We found that EXT1 was associated with KIF2C by PPI network analysis. And EXT1 may affect tumor survival by activating the Wnt signaling pathway By PPI network analysis, we found that EXT1 was associated with KIF2C. EXT1 may affect tumor survival by activating the Wnt signaling pathway (Kong et al., 2021), and it has also been demonstrated that KIF2C is activated by the Wnt signaling pathway and thus can promote liver cancer progression by activating mTORC1 signaling (Wei et al., 2020), so we speculate that EXT1 may influence KIF2C and thus promote tumor development.

Finally, we investigated the connection between KIF2C and anticancer drug resistance using CellMiner and found that KIF2C was negatively correlated with the sensitivity of Tamoxifen, Denileukin Diftitox Ontak, and Fluorouracil. Tamoxifen can be used to treat breast cancer (Shagufta and Ahmad, 2018), while Fluorouracil is one of the main components of many chemotherapy regimens and plays an important role in tumor chemotherapy (Ghafouri-Fard et al., 2021). High KIF2C expression was found to be associated with resistance to these drugs, and therefore, targeting KIF2C may have a role in improving the efficacy of chemotherapy treatment.

In summary, KIF2C was highly expressed in multiple tumor tissues and was significantly associated with poor prognosis. In tumor immunity, KIF2C was significantly associated with CAFs, TAMs, Tregs and MDSCs infiltrates in TME. KIF2C was significantly correlated with TMB, MSI, MRR and ICP genes in several tumors, and KIF2C expression was correlated with the sensitivity of some anticancer drugs. In tumor immunity, KIF2C was significantly associated with CAFs, TAMs, Tregs, and MDSCs infiltrating the TME. In several tumors, KIF2Cexpression was significantly correlated with TMB, MSI, MRR, and ICP genes, and KIF2C expression was correlated with the sensitivity of some anticancer drugs. Therefore, KIF2C is a prognostic biomarker linked to immunosuppression, targeting KIF2C may improve the outcome of immunotherapy.

## Data Availability

The original contributions presented in the study are included in the article/Supplementary Material, further inquiries can be directed to the corresponding author.

## References

[B1] An L. Zhang J. Feng D. Zhao Y. Ouyang W. Shi R. (2021). KIF2C Is a Novel Prognostic Biomarker and Correlated with Immune Infiltration in Endometrial Cancer. Stem Cells Int. 2021, 1–13. 10.1155/2021/1434856 PMC8510809 34650608

[B2] Barbieri I. Kouzarides T. (2020). Role of RNA Modifications in Cancer. Nat. Rev. Cancer 20, 303–322. 10.1038/s41568-020-0253-2 32300195

[B3] Chang L. Chang M. Chang H. M. Chang F. (2018). Microsatellite Instability: A Predictive Biomarker for Cancer Immunotherapy. Appl. Immunohistochem. Mol. Morphol. 26, e15–e21. 10.1097/PAI.0000000000000575 28877075

[B4] Chen F. Chandrashekar D. S. Varambally S. Creighton C. J. (2019). Pan-cancer Molecular Subtypes Revealed by Mass-Spectrometry-Based Proteomic Characterization of More Than 500 Human Cancers. Nat. Commun. 10, 5679. 10.1038/s41467-019-13528-0 31831737 PMC6908580

[B5] Dawson M. A. Kouzarides T. (2012). Cancer Epigenetics: From Mechanism to Therapy. Cell 150, 12–27. 10.1016/j.cell.2012.06.013 22770212

[B6] Devarkar S. C. Wang C. Miller M. T. Ramanathan A. Jiang F. Khan A. G. (2016). Structural Basis for m7G Recognition and 2′-O-Methyl Discrimination in Capped RNAs by the Innate Immune Receptor RIG-I. Proc. Natl. Acad. Sci. U. S. A. 113, 596–601. 10.1073/pnas.1515152113 26733676 PMC4725518

[B7] Eichenlaub-Ritter U. (2015). Microtubule Dynamics and Tumor Invasion Involving MCAK. Cell Cycle 14, 3353. 10.1080/15384101.2015.1093813 26375511 PMC4825562

[B8] Gabrilovich D. I. (2017). Myeloid-Derived Suppressor Cells. Cancer Immunol. Res. 5, 3–8. 10.1158/2326-6066.CIR-16-0297 28052991 PMC5426480

[B9] Ghafouri-Fard S. Abak A. Tondro Anamag F. Shoorei H. Fattahi F. Javadinia S. A. (2021). 5-Fluorouracil: A Narrative Review on the Role of Regulatory Mechanisms in Driving Resistance to This Chemotherapeutic Agent. Front. Oncol. 11, 658636. 10.3389/fonc.2021.658636 33954114 PMC8092118

[B10] Goldman M. J. Craft B. Hastie M. Repečka K. McDade F. Kamath A. (2020). Visualizing and Interpreting Cancer Genomics Data via the Xena Platform. Nat. Biotechnol. 38, 675–678. 10.1038/s41587-020-0546-8 32444850 PMC7386072

[B11] Ishikawa K. Kamohara Y. Tanaka F. Haraguchi N. Mimori K. Inoue H. (2008). Mitotic Centromere-Associated Kinesin Is a Novel Marker for Prognosis and Lymph Node Metastasis in Colorectal Cancer. Br. J. Cancer 98, 1824–1829. 10.1038/sj.bjc.6604379 18506187 PMC2410130

[B12] Jiang P. Lee W. Li X. Johnson C. Liu J. S. Brown M. (2018). Genome-Scale Signatures of Gene Interaction from Compound Screens Predict Clinical Efficacy of Targeted Cancer Therapies. Cell Syst. 6, 343–354. 10.1016/j.cels.2018.01.009 29428415 PMC5876130

[B13] Jones P. A. Ohtani H. Chakravarthy A. De Carvalho D. D. (2019). Epigenetic Therapy in Immune-Oncology. Nat. Rev. Cancer 19, 151–161. 10.1038/s41568-019-0109-9 30723290

[B14] Kong W. Chen Y. Zhao Z. Zhang L. Lin X. Luo X. (2021). EXT1 Methylation Promotes Proliferation and Migration and Predicts the Clinical Outcome of Non‐small Cell Lung Carcinoma via WNT Signalling Pathway. J. Cell Mol. Med. 25, 2609–2620. 10.1111/jcmm.16277 33565239 PMC7933929

[B15] Li T. Fan J. Wang B. Traugh N. Chen Q. Liu J. S. (2017). TIMER: A Web Server for Comprehensive Analysis of Tumor-Infiltrating Immune Cells. Cancer Res. 77, e108. –e110. 10.1158/0008-5472.CAN-17-0307 29092952 PMC6042652

[B16] Lin Q. Qi Q. Hou S. Chen Z. Jiang N. Zhang L. (2021). Activation of the TGF-β1/Smad Signaling by KIF2C Contributes to the Malignant Phenotype of Thyroid Carcinoma Cells. Tissue Cell 73, 101655. 10.1016/j.tice.2021.101655 34624565

[B17] Liu C.-J. Hu F.-F. Xia M.-X. Han L. Zhang Q. Guo A.-Y. (2018). GSCALite: a Web Server for Gene Set Cancer Analysis. Bioinformatics 34, 3771–3772. 10.1093/bioinformatics/bty411 29790900

[B18] Lu C. Guan J. Lu S. Jin Q. Rousseau B. Lu T. (2021). DNA Sensing in Mismatch Repair-Deficient Tumor Cells Is Essential for Anti-tumor Immunity. Cancer Cell 39, 96–108. 10.1016/j.ccell.2020.11.006 33338425 PMC9477183

[B19] Lucanus A. J. Yip G. W. (2018). Kinesin Superfamily: Roles in Breast Cancer, Patient Prognosis and Therapeutics. Oncogene 37, 833–838. 10.1038/onc.2017.406 29059174

[B20] Mao X. Xu J. Wang W. Liang C. Hua J. Liu J. (2021). Crosstalk between Cancer-Associated Fibroblasts and Immune Cells in the Tumor Microenvironment: New Findings and Future Perspectives. Mol. Cancer 20, 131. 10.1186/s12943-021-01428-1 34635121 PMC8504100

[B21] Mo S. Fang D. Zhao S. Thai Hoa P. T. Zhou C. Liang T. (2022). Down Regulated Oncogene KIF2C Inhibits Growth, Invasion, and Metastasis of Hepatocellular Carcinoma through the Ras/MAPK Signaling Pathway and Epithelial-To-Mesenchymal Transition. Ann. Transl. Med. 10, 151. 10.21037/atm-21-6240 35284538 PMC8904974

[B22] Nakamura Y. Tanaka F. Haraguchi N. Mimori K. Matsumoto T. Inoue H. (2007). Clinicopathological and Biological Significance of Mitotic Centromere-Associated Kinesin Overexpression in Human Gastric Cancer. Br. J. Cancer 97, 543–549. 10.1038/sj.bjc.6603905 17653072 PMC2360338

[B23] Petitprez F. Meylan M. de Reyniès A. Sautès-Fridman C. Fridman W. H. (2020). The Tumor Microenvironment in the Response to Immune Checkpoint Blockade Therapies. Front. Immunol. 11, 784. 10.3389/fimmu.2020.00784 32457745 PMC7221158

[B24] Quail D. F. Joyce J. A. (2013). Microenvironmental Regulation of Tumor Progression and Metastasis. Nat. Med. 19, 1423–1437. 10.1038/nm.3394 24202395 PMC3954707

[B25] Rath O. Kozielski F. (2012). Kinesins and Cancer. Nat. Rev. Cancer 12, 527–539. 10.1038/nrc3310 22825217

[B26] Reinhold W. C. Sunshine M. Liu H. Varma S. Kohn K. W. Morris J. (2012). CellMiner: A Web-Based Suite of Genomic and Pharmacologic Tools to Explore Transcript and Drug Patterns in the NCI-60 Cell Line Set. Cancer Res. 72, 3499–3511. 10.1158/0008-5472.CAN-12-1370 22802077 PMC3399763

[B27] Ritter A. Kreis N.-N. Louwen F. Wordeman L. Yuan J. (2016). Molecular Insight into the Regulation and Function of MCAK. Crit. Rev. Biochem. Mol. Biol. 51, 228–245. 10.1080/10409238.2016.1178705 27146484

[B28] Sadeghi Rad H. Monkman J. Warkiani M. E. Ladwa R. O’Byrne K. Rezaei N. (2021). Understanding the Tumor Microenvironment for Effective Immunotherapy. Med. Res. Rev. 41, 1474–1498. 10.1002/med.21765 33277742 PMC8247330

[B29] Sanhaji M. Friel C. T. Wordeman L. Louwen F. Yuan J. (2011). Mitotic Centromere-Associated Kinesin (MCAK): a Potential Cancer Drug Target. Oncotarget 2, 935–947. 10.18632/oncotarget.416 22249213 PMC3282097

[B30] Sha D. Jin Z. Budczies J. Kluck K. Stenzinger A. Sinicrope F. A. (2020). Tumor Mutational Burden as a Predictive Biomarker in Solid Tumors. Cancer Discov. 10, 1808–1825. 10.1158/2159-8290.CD-20-0522 33139244 PMC7710563

[B31] Shagufta Ahmad I. (2018). Tamoxifen a Pioneering Drug: An Update on the Therapeutic Potential of Tamoxifen Derivatives. Eur. J. Med. Chem. 143, 515–531. 10.1016/j.ejmech.2017.11.056 29207335

[B32] Shankavaram U. T. Varma S. Kane D. Sunshine M. Chary K. K. Reinhold W. C. (2009). CellMiner: a Relational Database and Query Tool for the NCI-60 Cancer Cell Lines. BMC Genomics 10, 277. 10.1186/1471-2164-10-277 19549304 PMC2709662

[B33] Shimo A. Tanikawa C. Nishidate T. Lin M.-L. Matsuda K. Park J.-H. (2007). Involvement of Kinesin Family Member 2C/mitotic Centromere-Associated Kinesin Overexpression in Mammary Carcinogenesis. Cancer Sci. 99, 62–70. 10.1111/j.1349-7006.2007.00635.x 17944972 PMC11158784

[B34] Shu Y. Cheng P. (2020). Targeting Tumor-Associated Macrophages for Cancer Immunotherapy. Biochimica Biophysica Acta (BBA) - Rev. Cancer 1874, 188434. 10.1016/j.bbcan.2020.188434 32956767

[B35] Warde-Farley D. Donaldson S. L. Comes O. Zuberi K. Badrawi R. Chao P. (2010). The GeneMANIA Prediction Server: Biological Network Integration for Gene Prioritization and Predicting Gene Function. Nucleic Acids Res. 38, W214–W220. 10.1093/nar/gkq537 20576703 PMC2896186

[B36] Wei S. Dai M. Zhang C. Teng K. Wang F. Li H. (2020). KIF2C: a Novel Link between Wnt/β-Catenin and mTORC1 Signaling in the Pathogenesis of Hepatocellular Carcinoma. Protein Cell 12, 1–22. 10.1007/s13238-020-00766-y PMC8464548 32748349

[B37] Wolf D. Sopper S. Pircher A. Gastl G. Wolf A. M. (2015). Treg(s) in Cancer: Friends or Foe?: THE AMBIGUOUS ROLE of TREG IN CANCER. J. Cell. Physiol. 230, 2598–2605. 10.1002/jcp.25016 25913194

[B38] Yang J. Wu Z. Yang L. Jeong J.-H. Zhu Y. Lu J. (2022). Characterization of Kinesin Family Member 2C as a Proto-Oncogene in Cervical Cancer. Front. Pharmacol. 12, 785981. 10.3389/fphar.2021.785981 35153749 PMC8828917

[B39] Zhang B. Wu Q. Li B. Wang D. Wang L. Zhou Y. L. (2020). m6A Regulator-Mediated Methylation Modification Patterns and Tumor Microenvironment Infiltration Characterization in Gastric Cancer. Mol. Cancer 19, 53. 10.1186/s12943-020-01170-0 32164750 PMC7066851

[B40] Zhang X. Huang T. Li Y. Qiu H. (2021). Upregulation of THBS1 Is Related to Immunity and Chemotherapy Resistance in Gastric Cancer. IJGM 14, 4945–4957. 10.2147/IJGM.S329208 PMC8407783 34475782

