# Supplementary material for: KIF2C is a Biomarker Correlated With Prognosis and Immunosuppressive Microenvironment in Human Tumors
Source: Front Genet. 2022 May 24;13:891408. doi: 10.3389/fgene.2022.891408 (PMC9171145; doi:10.3389/fgene.2022.891408)
Supplement: Supplementary file 4 [file DataSheet1.PDF]

Supplementary Material

1 Supplementary Figures

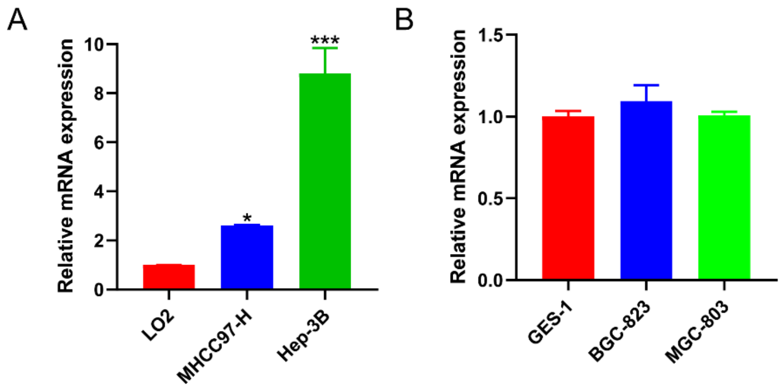

Supplementary Figure 1. Expression of KIF2C in cell lines. (A) Expression of KIF2C in hepatocellular carcinoma cell lines. (B) Expression of KIF2C in gastric cancer cell lines

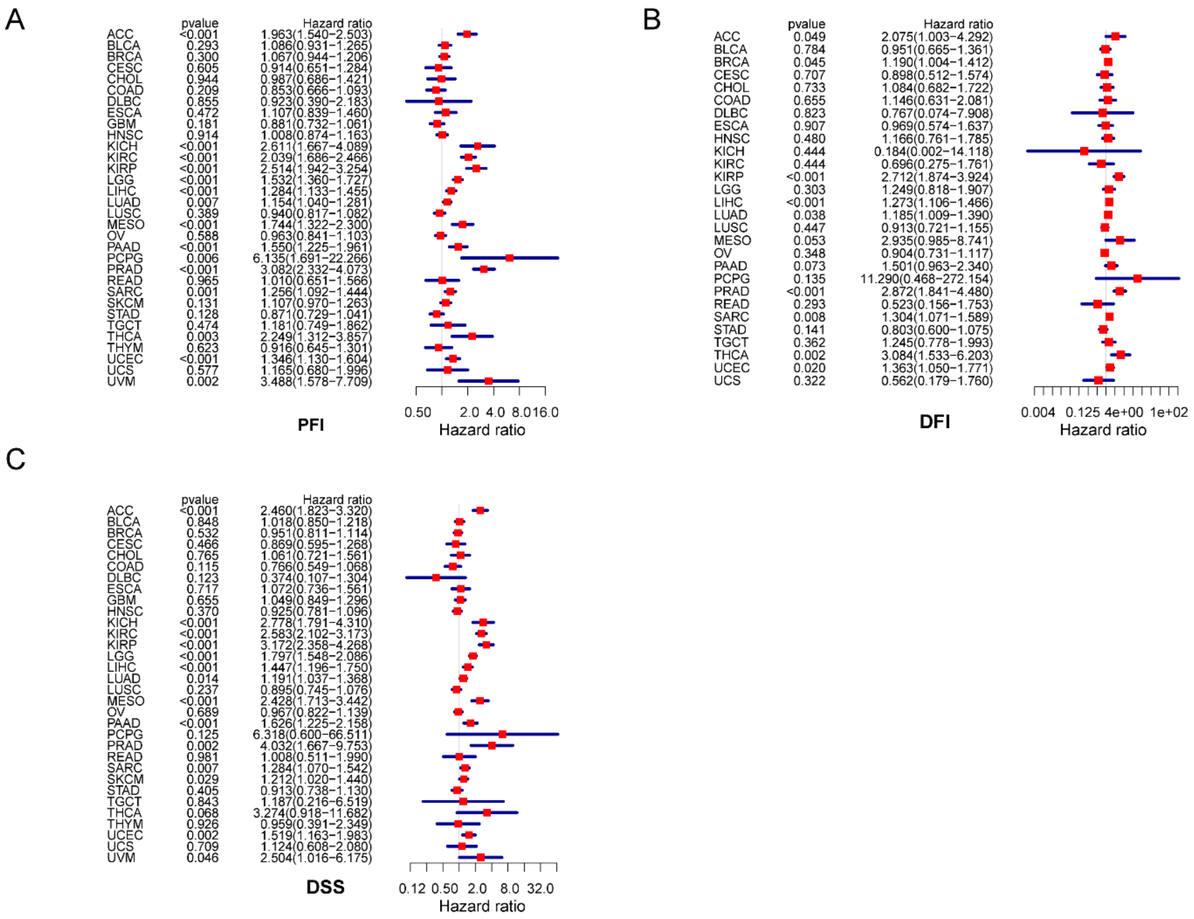

Supplementary Figure 2. The correlation between KIF2C expression and prognosis for PFI, DFI, DSS in pan-cancer. (A) The correlation between KIF2C expression and PFI in different cancer types of TCGA. (B) The correlation between KIF2C expression and DFI in different cancer types of TCGA. (C) The correlation between KIF2C expression and DSS in different cancer types of TCGA. The red part represents the risk ratio.

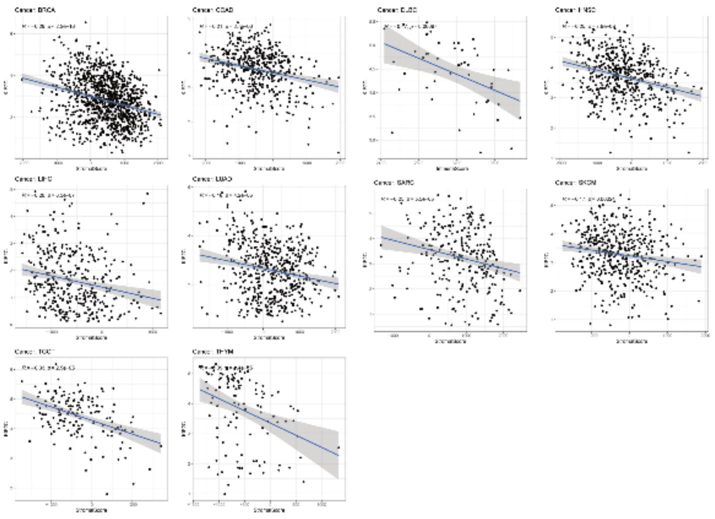

Supplementary Figure 3. Correlation of KIF2C with stromal scores of BRCA, COAD, HNSC, LIHC, LUAD, SARC, SKCM, TGCT, THYM and immune scores of DLBC.

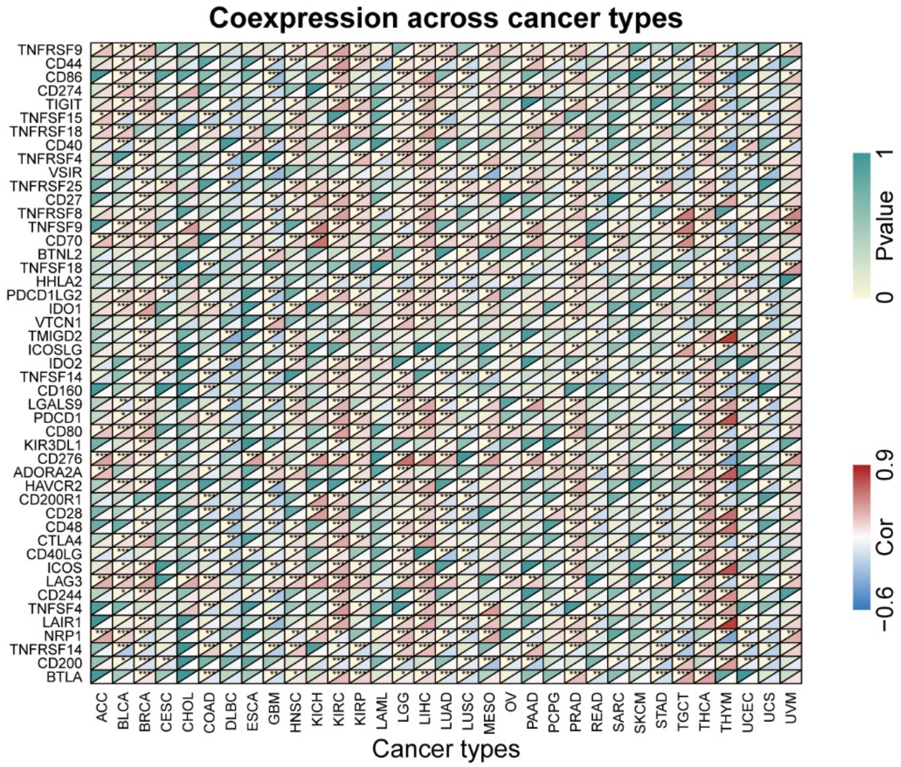

Supplementary Figure 4. The correlations between KIF2C expression and immune checkpoint genes in pan-cancer. * p<0.05, ** p<0.01, *** p < 0.001， ****p<0.0001.

2 Supplementary Tables

Supplementary Table1: The abbreviations and meanings of the 33 tumor types.

Supplementary Table2: The correlation between KIF2C expression and immune markers in KIRC, LGG, LIHC and TYHM.

Supplementary Table3: CARE database analysis results.
